# Combined exposure to cigarette smoke and nontypeable *Haemophilus influenzae* drives development of a COPD phenotype in mice

**DOI:** 10.1186/1465-9921-15-11

**Published:** 2014-02-04

**Authors:** Shyamala Ganesan, Adam T Comstock, Brenton Kinker, Peter Mancuso, James M Beck, Uma S Sajjan

**Affiliations:** 1 Department of Pediatrics and Communicable Diseases, University of Michigan, 1150 W. Medical Center Dr., Ann Arbor, MI 48109-5688, USA; 2 School of Public Health, University of Michigan, Ann Arbor, MI, USA; 3 Division of Pulmonary Sciences and Critical Care Medicine, Department of Medicine, University of Colorado, Aurora, CO, USA; 4 Veterans Affairs, Eastern Colorado Health Care System, Denver, CO, USA

**Keywords:** COPD exacerbation, Viral infection, Airway epithelium, COPD pathology, Emphysema

## Abstract

**Background:**

Cigarette smoke (CS) is the major etiologic factor of chronic obstructive pulmonary disease (COPD). CS-exposed mice develop emphysema and mild pulmonary inflammation but no airway obstruction, which is also a prominent feature of COPD. Therefore, CS may interact with other factors, particularly respiratory infections, in the pathogenesis of airway remodeling in COPD.

**Methods:**

C57BL/6 mice were exposed to CS for 2 h a day, 5 days a week for 8 weeks. Mice were also exposed to heat-killed non-typeable *H. influenzae* (HK-NTHi) on days 7 and 21. One day after the last exposure to CS, mice were sacrificed and lung inflammation and mechanics, emphysematous changes, and goblet cell metaplasia were assessed. Mice exposed to CS or HK-NTHi alone or room air served as controls. To determine the susceptibility to viral infections, we also challenged these mice with rhinovirus (RV).

**Results:**

Unlike mice exposed to CS or HK-NTHi alone, animals exposed to CS/HK-NTHi developed emphysema, lung inflammation and goblet cell metaplasia in both large and small airways. CS/HK-NTHi-exposed mice also expressed increased levels of mucin genes and cytokines compared to mice in other groups. CS/HK-NTHi-exposed mice infected with RV demonstrated increased viral persistence, sustained neutrophilia, and further increments in mucin gene and chemokine expression compared to other groups.

**Conclusions:**

These findings indicate that in addition to CS, bacteria may also contribute to development of COPD, particularly changes in airways. Mice exposed to CS/HK-NTHi are also more susceptible to subsequent viral infection than mice exposed to either CS or HK-NTHi alone.

## Background

Chronic obstructive pulmonary disease (COPD) is the fourth leading cause of death world-wide, and the prevalence of COPD is increasing globally [1]. COPD is characterized by airflow limitation that is progressive and is usually irreversible. Small airway remodeling, including narrowing of airways due to peribronchiolar fibrosis, goblet cell metaplasia and excessive mucus production, is now accepted as an important cause of airflow obstruction in COPD [2]. In addition, lung parenchyma is destroyed by proteolytic damage (emphysema), reducing the elasticity and gas-exchange surface area of the lung. Acute exacerbations further enhance airways obstruction and accelerate progression of lung disease in these patients (reviewed in [3]). However, the underlying mechanisms for these changes are not well understood, partly because small-animal models do not recapitulate all the typical features of human COPD.

COPD has been modeled in mice by administration of proteases, lipopolysaccharide (LPS), chemicals and cigarette smoke (CS). In particular, CS has been used extensively to investigate mechanisms of COPD pathogenesis since it is the major risk factor in the development of COPD [3]. While whole body exposure to CS for a short-term (3 days to 4 weeks) has been useful in evaluating the mechanisms of CS-induced acute lung inflammation and defective innate immune responses to subsequent infections [4-7], long-term exposure to CS for periods up to 6 months has been employed to understand the mechanisms of emphysema development [8-11]. However, neither of these models show changes in small airways, which plays a major role in the development of airflow limitation in COPD implying that other factors in addition to CS are required to mimic COPD lung disease in mice [12].

Although CS exposure is the key insult in the pathogenesis of COPD, only 25 to 35% of smokers develop COPD [13,14] suggesting the contribution of other factors, such as genetic background, concurrent respiratory infections, and aberrant host responses in the development of COPD. Respiratory pathogens including bacteria, viruses and fungi are often present in the airways of COPD patients and therefore it is plausible that respiratory pathogens or their products such as enterotoxin, endotoxin, viral RNA may contribute to disease pathogenesis [15-17]. For example Kang *et al.* demonstrated that CS synergizes with synthetic double stranded (ds) RNA, a viral RNA mimetic to induce enhanced inflammatory and emphysematous changes, and airway fibrosis in the mouse lungs [17]. However these mice did not develop goblet cell metaplasia, increased mucus production or airways obstruction, which are also important features of COPD. Similar results were observed when mice were infected with influenza virus instead of treating with dsRNA. Enterotoxin B isolated from *S. aureus* was shown to exacerbate CS-induced inflammatory changes in mouse lungs and induce goblet cell metaplasia and formation of lymphoid aggregates, but these mice did not develop emphysema. Chronic exposure of mice to lysates of non-typeable *H. influenzae* (NTHi) was shown to induce airway inflammation but not emphysema or airway remodeling [18]. Endotoxin, a bacterial cell wall component is present in abundant amounts as a contaminant in CS [19,20] and prolonged intratracheal exposure of mice to endotoxin, (twice week for 3 months) induces lung inflammation, and changes in both parenchyma and airways which persists up to 8 weeks [21]. We demonstrated that exposure of mice once a week to combination of elastase and endotoxin for 4 weeks induces all the features of COPD [22]. The latter three models although exhibit features of COPD and indicate involvement of bacterial factors in COPD pathogenesis, these models may not be representative of CS-induced changes. Based on these observations, in the present study we examine a novel concept that in addition to CS, exposure to bacteria is required to induce typical features of COPD including emphysema, airway remodeling, and lung inflammation in mice. Since NTHi is frequently isolated from clinically stable COPD patients as well as during exacerbations [23], we used NTHi in the present study. We also evaluated susceptibility to rhinovirus (RV) infection in this model, because RV is associated with virally mediated exacerbations of COPD and sometimes leads to progression of lung disease [24,25].

## Methods

### Animals and treatment

Eight to ten week old C57BL/6 mice (Charles River Laboratories, Wilmington, MA) were exposed to CS as described previously [26]. Briefly mice were exposed to smoke from standardized 3R4F research cigarettes (University of Kentucky, Lexington, KY) generated by a TE-2 cigarette smoking machine (Teague Enterprises, Woodland, CA). This device is set up to provide a mixture of mainstream and sidestream smoke. Animals were exposed to CS for 2 h/day, 5 days/week for 8 consecutive weeks in a 54-L glass and Plexiglas whole-body exposure chamber with an electric fan for chamber mixing in standard mouse caging units with wire cage tops, with water available *ad libitum*. The mean concentration of particulates collected during a 2-h exposure was 9.28 ± 1.45 mg/day. Control mice were exposed to room air (RA) under similar conditions. Heat-killed (HK) NTHi was administered into the lungs on days 7 and 21 by intranasal route. Three days after the last exposure to CS, lungs were lavaged [27] to determine total and differential cell counts and to measure cytokines, were fixed in formalin and embedded in paraffin [22] to evaluate morphology, or were homogenized in PBS to isolate total RNA. In additional experiments, mice were inoculated intranasally with RV or with a sham preparation, and sacrificed 4 days post infection. All experiments were approved by the Animal Care and Use Committee of the University of Michigan and Veterans Affairs, Ann Arbor Medical Center Animal Care Committee.

### Heat killed bacteria

NTHi strain, 5P54H1 was isolated from a COPD patient and was kindly provided by Dr. Timothy Murphy, University of Buffalo. Bacteria were cultured on chocolate agar, suspended in PBS to density of 1 × 10^9^ CFU/ml and incubated at 80°C for 30 min to kill the bacteria. Mice received 50 μl of heat-killed (HK) NTHi or PBS intranasally [28].

### Rhinovirus and infection

Stocks of RV1B were prepared by infecting HeLa cells with RV1B and subjecting HeLa cell supernatants to ultrafiltration and then viral titer was determined as described previously [22,29]. Similarly concentrated and purified cell supernatants from uninfected HeLa cells were used as sham controls. At the end of 8 weeks of exposure to CS or RA and +/- HK-NTHi, mice were infected with RV or an equal volume of sham preparation by intranasal route as described previously [22] and sacrificed 4 days post infection.

### Bronchoalveolar Lavage (BAL)

After relevant treatment, mice were sacrificed and BAL was performed by instilling 1 ml PBS containing 5 mM EDTA 10 times [27]. The first wash was centrifuged and supernatant was retained for determination of cytokines by ELISA. Cells from the first wash were combined with the cells from rest of the washes and suspended in 1 ml of PBS and numbers of cells were counted to determine total cells. Cytospins prepared from BAL cells were stained with Diff-Quick and differential counts were determined by counting a minimum of 200 cells.

### RNA isolation and qPCR

Lungs were collected under aseptic conditions after relevant treatment and homogenized in 2 ml PBS. An aliquot of lung homogenate was immediately mixed with TRIZOL and total RNA was then purified by using RNeasy miRNA kit (Qiagen, Alameda, CA). Total RNA was reverse transcribed to first strand cDNA using Taqman reverse transcription kit (Applied Biosystems Life Technologies, Carlsbad, CA). cDNA was then used to determine the mRNA expression of IP-10, Muc5B, Muc5AC, Gob5 and G3PDH (house-keeping gene) by quantitative qPCR using gene specific primers and Syber green PCR mix. All primers were designed and purchased from IDT (Coralville, IA). To detect viral RNA, total RNA isolated from the lungs was subjected quantitative Taqman qPCR as described previously [30] and expressed as number of vRNA copies per 10 μg of total RNA.

### Infectious viral load

Lung homogenates were used to determine the infectious viral load by plaque assay as described [22].

### Histology and morphometry

Lungs were inflation fixed at a constant pressure of 30 cm.H_2_O for 30 min. Lungs were embedded in paraffin, and 5 μ thick sagittal sections were stained with hematoxylin and eosin (H & E) to assess histology or periodic acid-Schiff (PAS) to detect goblet cells. Alveolar chord length was determined as described previously [22,31]. Briefly, sagittal sections obtained at 5 mm intervals through the depth of the lungs were stained with H & E and the diameters of the air spaces measured in at least 10 random areas using NIH image J analysis software. Sagittal sections obtained at 5 mm intervals were also stained with PAS and the number of PAS positive cells per 100 μM basement membrane lengths was counted in random fields. Airways with perimeter of ≤1 mM were considered as small airways. Airways with cartilage were considered as large airways [22].

### Lung mechanics and airways resistance

Dynamic lung elastance and compliance, pressure-volume relationship in mice exposed to RA, CS, RA/HK-NTHi or CS/HK-NTHi, were measured as described previously using a miniature computerized flexivent ventilator (Scireq, Canada) [22]. Airways responsiveness to increasing dose of methacholine challenge following RV infection was measured by plethysmography [30].

### ELISA

Bronchoalveolar lavage fluid was centrifuged and the supernatant was used for assessing the levels of chemokines and cytokines by ELISA.

### Statistical Analysis

Results are expressed as means ± SD or mean with range of data. Data were analyzed by using SigmaStat statistical software (Systat Software, San Jose, CA). One- or two-way ANOVA with Tukey’s range test, ANOVA on ranks with Friedman’s test or unpaired *t* test was performed as appropriate to compare groups and a *P* value ≤ 0.05 was considered significant.

## Results

### Lung histology

**Figure 1:**
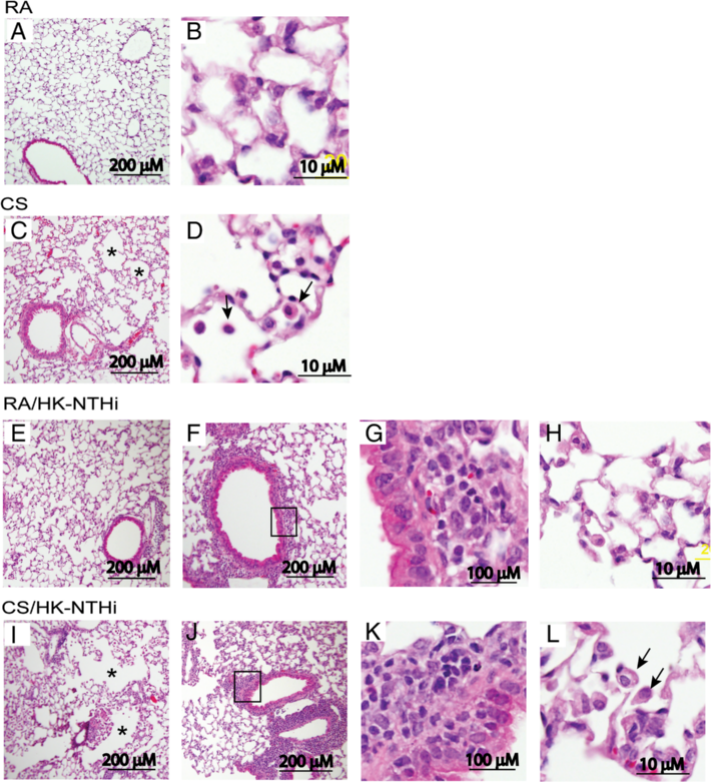
**Histological evaluation of lungs.** Lungs harvested from mice exposed to RA **(A and B)**, CS **(C and D)**, HK-NTHi **(E to H)** or CS/HK-NTHi **(I to L)** were inflation fixed in formalin, and embedded in paraffin. Five micron thick sections were stained with H&E and observed under light microscope. Arrows in D and L represent infiltrated inflammatory cells in alveolar space. G and K represent magnified view of marked area in F and J respectively and show accumulated macrophages in peribronchiolar area. Asterisks in D and I represents emphysema. Images are representative of 3–4 mice per group.

**Figure 2:**
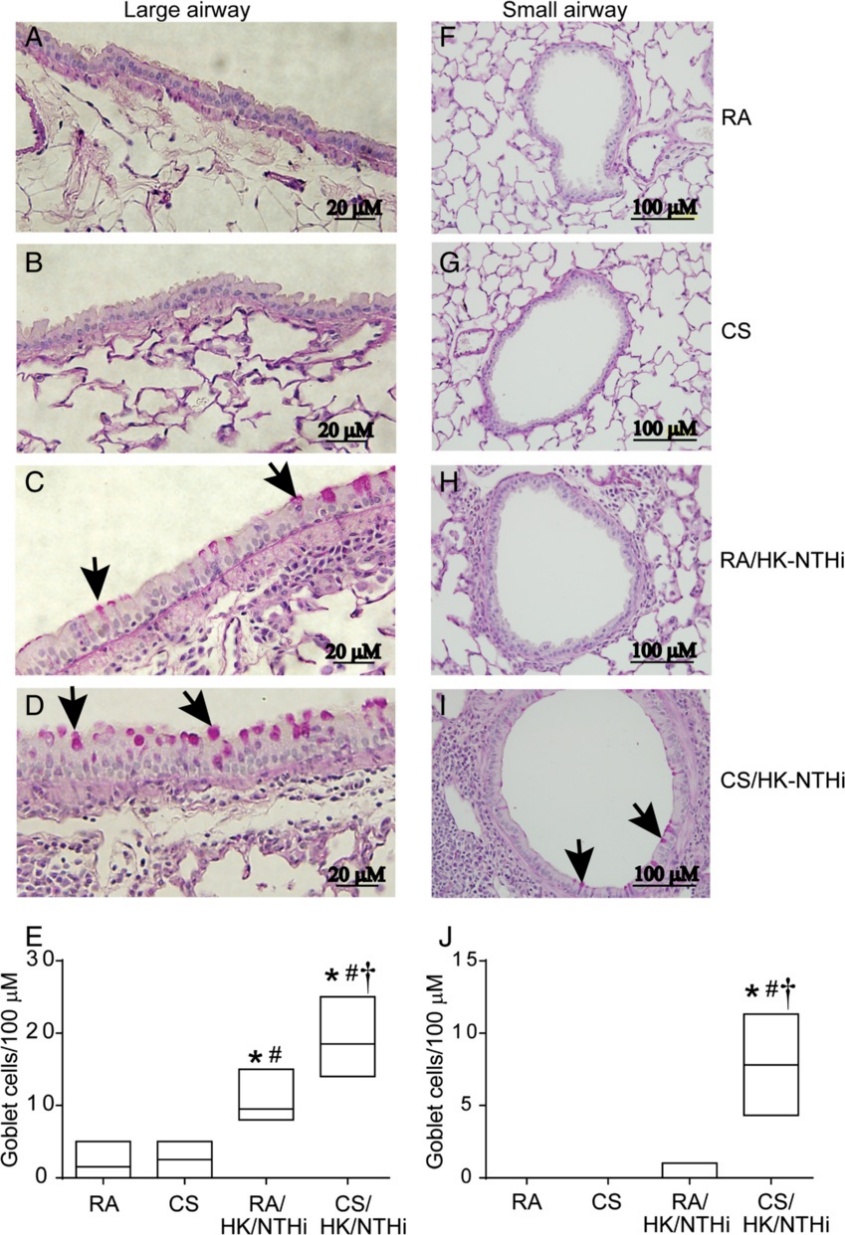
**PAS staining of lung sections.** Paraffin lung sections were stained with PAS to determine goblet cell metaplasia in large and small airways. **A to D**, represents large airways and **F to I** represents small airways from mice exposed to RA, CS, HK-NTHi and CS/HK-NTHi respectively. Arrows in C, D and I represents cells positive for PAS, a marker of goblet cells. Number of PAS positive cells in large **(E)** and small **(J)** airway epithelia were counted in at least 10 random fields per slide and expressed as number of goblet cells/100 μM basement membrane length. Data represents median with range calculated from 5–6 mice (* different from RA-exposed mice, p ≤ 0.05, ANOVA on ranks; # different from CS-exposed mice, p ≤ 0.05, ANOVA on ranks; † different from HK-NTHi-exposed mice, p ≤ 0.05, ANOVA on ranks). Airway epithelial hyperplasia is observed in large airways of HK-NTHi **(C)** and both large **(D)** and small **(H)** airways of CS/HK-NTHi exposed mice. Images are representative of 5–6 mice per group.

Lung sections from mice exposed to room air (RA), RA/HK-NTHi, CS, CS-HK-NTHi were evaluated by light microscopy. RA-exposed mice showed normal morphology with baseline air space size and no appreciable inflammation (Figure 1A and 1B). CS-exposed mice showed mild lung inflammation, enlarged airspaces in a patchy distribution, and accumulated inflammatory cells (possibly macrophages) in the airspaces (Figure 1C and 1D). Mice exposed to HK-NTHi showed no enlarged airspaces, but demonstrated peribronchiolar inflammation (Figure 1E to 1H). Mice treated with a combination of CS and HK-NTHi showed enlarged airspaces and mild lung inflammation (Figure 1I), as well as accumulation of inflammatory cells in peribronchiolar and perivascular areas (Figure 1J and 1K) and in the airspaces (Figure 1L). Compared to RA- or CS-exposed mice, both HK-NTHi and CS/HK-NTHi-exposed mice showed increases in the numbers of PAS positive cells (indicative of goblet cell metaplasia) in large airway epithelia, however it was more pronounced in CS/NK-NTHi-exposed mice (Figure 2A to 2D). Mice in CS/HK-NTHi group, but not animals in other groups showed increased numbers of PAS positive cells in small airway epithelia (Figure 2E to 2H). Mice exposed to HK-NTHi or CS/HK-NTHi also showed airway epithelial hyperplasia in both small and large airways, but it was more prominent in CS/HK-NTHi-exposed mice.

### Morphometry and lung mechanics

**Figure 3:**
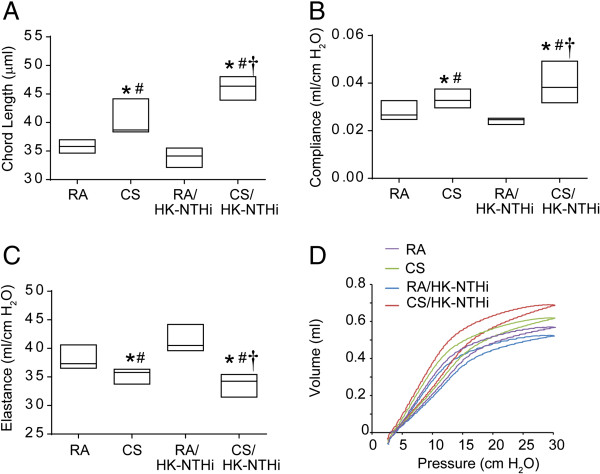
**Morphometry and lung mechanics. (A)** Lungs of mice exposed to RA, CS, HK-NTHi or CS/HK-NTHi were inflated to an identical pressure, processed for paraffin embedding and sections at different depth were stained with H & E. The diameters of the air spaces were measured in at least 10 random fields per slide.. Mice were anesthetized and dynamic compliance **(B)** and elastance **(C)** and pressure-volume relationships **(D)** were measured using Flexivent system. Data in A to C represent median with range calculated from 5 to 6 mice per group (* different from RA-exposed mice, p ≤ 0.05, ANOVA on ranks; # different from RA/HK-NTHi-exposed mice, p ≤ 0.05, ANOVA on ranks; † different from CS-exposed mice, p ≤ 0.05, ANOVA on ranks).

Morphometric analysis was performed to determine the mean airspace chord length. Compared to RA-exposed mice both CS- and CS/HK-NTHi-exposed mice showed small but significant increases in chord length (RA vs CS, p = 0.045; RA vs CS/HK-NTHi, p = 0.002) (Figure 3A), but mice exposed to HK-NTHi did not. The changes in chord length in mice exposed to combination of CS and HK-NTHi were higher than in mice exposed to CS alone (p = 0.031).

We assessed elasticity of the lungs by determining total respiratory system compliance, elastance, and the pressure-volume (P-V) relationships during inflation. Compared to RA- or HK-NTHi-exposed mice, CS and CS/HK-NTHi- exposed mice showed increased compliance (CS vs RA, p = 0.047; CS vs HK-NTHi, p = 0.039; CS/HK-NTHi vs RA, p = 0.022; CS/HK-NTHi vs HK-NTHi, p = 0.029) (Figure 3B). Conversely, a significant decreases in elastance (Figure 3C) were observed in CS or CS/HK-NTHi group compared to mice exposed to RA or HK-NTHi (CS vs RA, p = 0.038; CS vs HK-NTHi, p = 0.025, CS/HK-NTHi vs RA, p = 0.011; CS/HK-NTHi vs HK-NTHi, p = 0.007). However, the changes in both compliance and elastance were significantly higher in CS/HK-NTHi-exposed mice than in CS-exposed mice (compliance, p = 0.034 and elastance, p = 0.021). Consistent with these observations, we also observed slightly upward and leftward shifts in the P-V relationships in CS- and CS/HK-NTHi-exposed mice compared to mice in other two groups, demonstrative of reduced elastic recoil. However, the changes in P-V relationship were more pronounced in CS/HK-NTHi-exposed mice (Figure 3D).

### Airways inflammation and mucin gene expression

**Figure 4:**
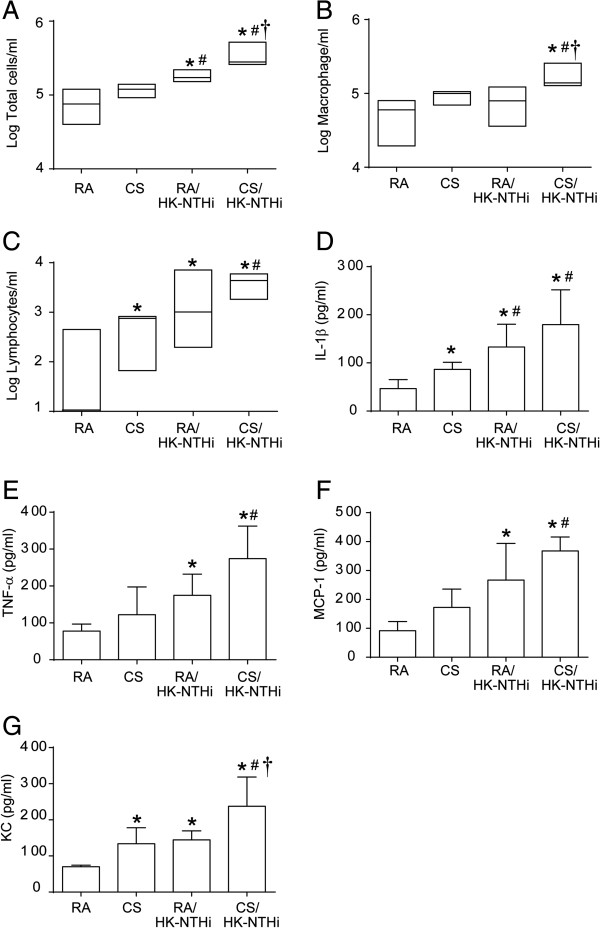
**Lung inflammation.** Bronchoalveolar lavage from RA, CS, HK-NTHi or CS/HK-NTHi mice was centrifuged and the pellet was used to determine total **(A)** and differential cell **(B** and **C)** counts. Supernatant was used to determine levels of IL-1β **(D)**, TNF-α **(E)**, MCP-1 **(F)** and KC **(G)** by ELISA. Data in A to C and D to G respectively represent median with range and mean ± SD calculated from 5–6 mice per group (* different from RA-exposed mice, p ≤ 0.05, ANOVA on ranks in A to C and ANOVA in D to G; # different from CS-exposed mice, p ≤ 0.05, ANOVA on ranks in A to C and ANOVA in D to G; † different from HK-NTHi-exposed mice, ANOVA on ranks in A to C and ANOVA in D to G).

To assess lung inflammation, we performed total and differential cell counts and quantified levels of IL-1β, TNF-α, KC and MCP-1 by ELISA. Compared to mice exposed to RA or CS, mice in HK-NTHi and CS/HK-NTHi groups showed a significantly higher numbers of total cells (RA vs HK-NTHi, p = 0.043; CS vs HK-NTHi, p = 0.048; RA vs CS/HK-NTHi p = 0.013; CS vs CS/HK-NTHi, p = 0.025) with the largest increase observed in the CS/HK-NTHi group (HK-NTHi vs CS/HK-NTHi, p = 0.0103) (Figure 4A). Both macrophages (Figure 4B) and lymphocytes were higher (Figure 4C) in the airways of CS/HK-NTHi than in RA-exposed mice (p = 0.027 and p = 0.031 respectively). Only the number of lymphocytes increased significantly in CS, HK-NTHi-exposed mice compared to RA-exposed mice (p = 0.049 and p = 0.043 respectively). There were no significant differences in numbers of neutrophils among the groups (data not shown). We then examined the levels of cytokines in the lavage fluid (Figure 4D to 4G). Compared to mice exposed to RA, mice in all other groups showed more IL-1β (RA vs CS, p = 0.043; RA vs HK-NTHi, p = 0.042; RA vs CS/HK-NTHi, p = 0.037) and KC levels (RA vs CS, p = 0.010; RA vs HK-NTHi, p = 0.007; RA vs CS/HK-NTHi, p = 0.023). Mice in HK-NTHi or CS/HK-NTHi groups, but not in the CS group showed higher levels of MCP-1 (RA vs HK-NTHi, p = 0.042; RA vs CS/HK-NTHi, p = 0.001) and TNF-α (RA vs HK-NTHi, p = 0.049; RA vs CS/HK-NTHi, p = 0.02) than the RA group. However, mice in the CS/HK-NTHi group showed the highest increases in the levels of all the cytokines measured.

**Figure 5:**
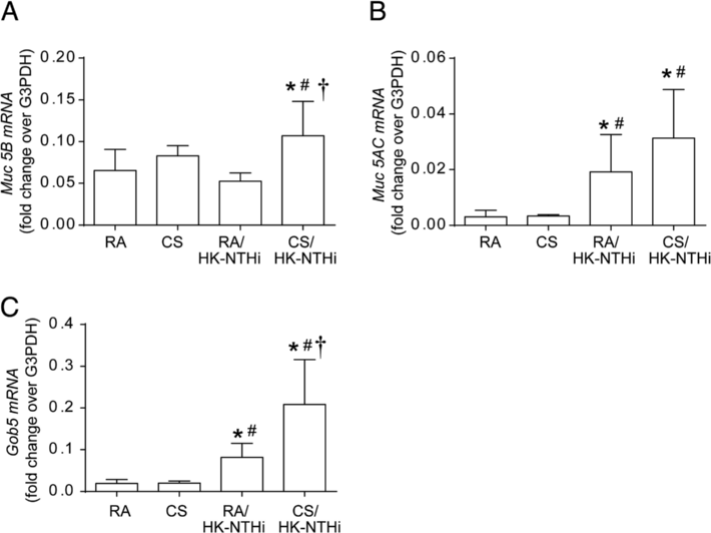
**Expression of mucin genes, *Muc5B *and *Muc5AC *and *Gob5*.** Total RNA was isolated from the lungs of mice exposed to RA, CS, HK-NTHi or CS/HK-NTHi and mRNA expression of *Muc5B***(A)**, *Muc5AC***(B)** and *Gob5***(C)** was assessed by qPCR and expressed as fold change over house-keeping gene, G3PDH. Data represents mean ± SD calculated from 6 mice per group (* different from RA-exposed mice, p ≤ 0.05, ANOVA; # different from CS-exposed mice, p ≤ 0.05, ANOVA; † different from HK-NTHi-exposed mice, p ≤ 0.05, ANOVA).

We then measured the expression of mucin genes, *Muc5B* and *Muc5AC* and a calcium-activated chloride channel that is thought to regulate mucus production and/or secretion, *Gob5* (*mclca3*) [32] by qPCR. The expression of *Muc5AC* and *Gob5*, but not *Muc5B* was increased in HK-NTHi-exposed mice compared to RA-exposed, but not in CS-exposed mice (*Muc5AC-* RA vs HK-NTHi, p = 0.041; *Gob5-* RA vs HK-NTHi, p = 0.038) (Figure 5A to 5C). In contrast, CS/HK-NTHi-exposed mice showed increases in *Muc5B* (p = 0.041) and *Muc5AC* (p = 0.045) as well as *Gob5* (p = 0.039) compared to RA-exposed mice. Again CS/HK-NTHi mice showed the largest increases in expression in all three genes.

### Susceptibility to RV infection

**Figure 6:**
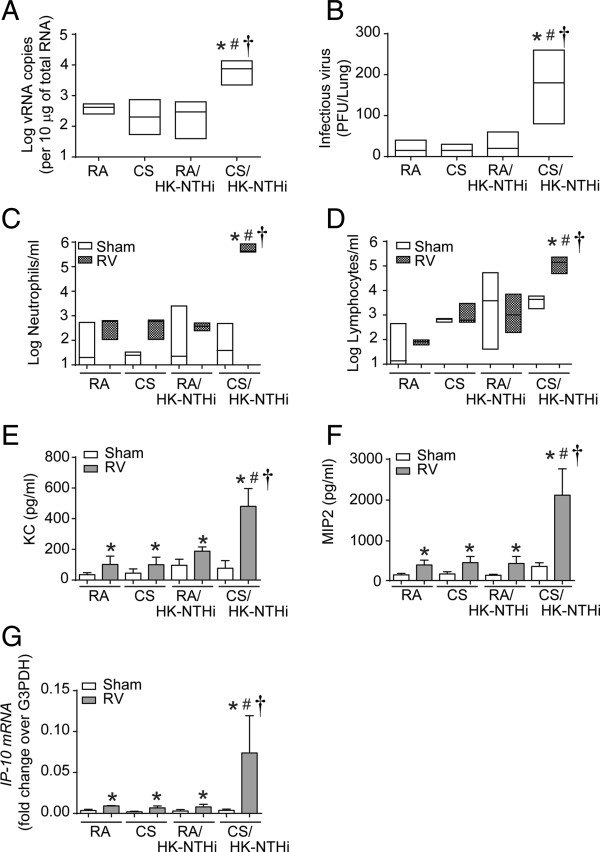
**Assessment of susceptibility of RA, CS, HK-NTHi or CS/HK-NTHi-exposed mice to RV infection.** RA, CS, HK-NTHi or CS/HK-NTHi-exposed mice were either infected with RV or equal volume of sham and mice were sacrificed 4 days post-infection. **(A)** Total RNA was isolated from whole lungs and viral RNA (vRNA) copy number was determined by quantitative qPCR and expressed vRNA copy number per 10 μg of total RNA. **(B)** Lung homogenates were used to determine infectious viral load by assessing number of plaque forming units (PFU) per lung. **(C and D)**. Cells from bronchoalveolar lavage were used to determine number of neutrophils and lymphocytes and expressed as cells/ml of BAL. Data in A to D represent median with range calculated from 5 to 6 mice per group (* different from RA-exposed mice, p ≤ 0.05, ANOVA on ranks; # different from CS-exposed mice, p ≤ 0.05, ANOVA on ranks; † different from HK-NTHi-exposed mice, p ≤ 0.05, ANOVA on ranks). **(E and F)** Supernatants from bronchoalveolar lavage were used to determine KC and MIP2 levels. **(G)** Total lung RNA was reverse transcribed and subjected to qPCR to determine mRNA levels of IP-10 and expressed as fold change over house-keeping gene, G3PDH. Data in E to G represents mean ± SD calculated from 5–6 mice per group (* different from respective sham controls, p ≤ 0.05, ANOVA; # different from CS-exposed RV-infected mice, p ≤ 0.05, ANOVA; † different from HK-NTHi-exposed RV-infected mice, p ≤ 0.05, ANOVA).

At the end of 8 weeks of exposure to CS, mice were infected with RV or sham, sacrificed after 4 days and examined for viral RNA, chemokine expression, neutrophil infiltration and mucin gene expression. CS/HK-NTHi-exposed mice infected with RV showed one log higher vRNA than similarly infected animals in other groups (p = 0.024) (Figure 6A). CS/HK-NTHi mice also showed higher infectious viral load than mice in other groups (p = 0.007), but it was only 1 to 2 × 10^2^ PFU/lung (Figure 6B). Despite having low levels of infectious viral load, the CS/HK-NTHi group showed remarkably higher neutrophil (p = 0.001) and lymphocyte (p = 0.009) infiltration compared to the sham infected group (Figure 6C and 6D). Consistent with the cellular infiltration, CS/HK-NTHi mice infected with RV also showed persistent increases in KC (p = <0.001), MIP-2 (p = 0.001) and IP-10 (0.022), compared to sham infected mice (Figure 6E- 6G). RV infected mice in other groups showed very small increases in infiltrated neutorphils, lymphocytes and cytokine levels over their respective sham-infected controls (p values ranged between 0.039 to 0.05 in these animals).

**Figure 7:**
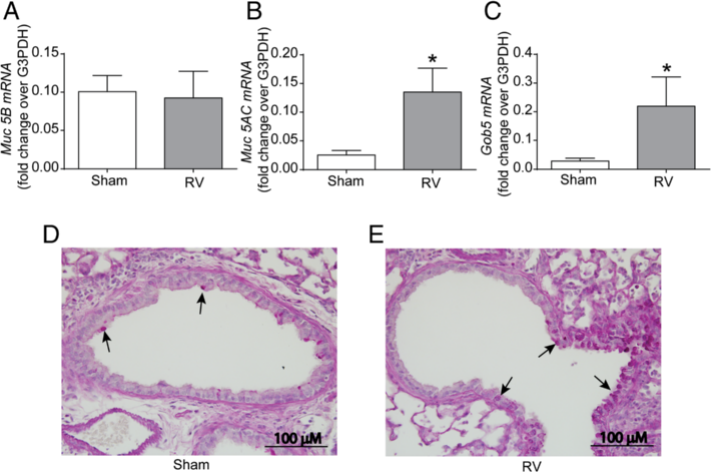
**Expression of mucin genes and goblet cell metaplasia in CS/HK-NTHi-exposed mice following RV-infection.** CS/HK-NTHi-exposed mice were infected with RV or equal volume of sham and sacrificed 4 days later. **(A to C)** Total lung RNA was isolated, reverse transcribed and subjected to qPCR using gene specific primers and expressed as fold change over house-keeping gene, G3PDH. Data in E to G represents mean ± SD calculated from 5–6 mice per group (* different from sham infected mice, p ≤ 0.05, unpaired *t* test). **(D and E)** Paraffin lung sections from sham- or RV-infected animals were stained with PAS. Arrows point to PAS positive goblet cells. Images are representative of 3 animals per group.

To assess the effects of RV infection on airways obstruction, we examined the expression of mucin genes, *Gob5*, goblet cell metaplasia in small airways, and responses to methacholine challenge. Irrespective of infection, mice exposed to RA, CS or RA/HK-NTHi did not show changes in the expression of *Muc5B*, *Muc5AC* or *Gob5* (data not shown). In contrast, CS/HK-NTHi-exposed mice infected with RV showed additional increases in the expression of *Muc5AC* (p = 0.041) and *Gob5* (p = 0.003), but not *muc2B* compared to similarly-exposed sham-infected mice (Figure 7A to 7C). CS/HK-NTHi-exposed mice infected with RV also showed a further increment in PAS-positive cells in the small airways compared to sham-infected mice, implying that enhanced mucus expression may be due to increased goblet cell metaplasia in these mice (Figure 7D and 7E). In contrast, mice exposed to RA, CS or RA/HK-NTHi infected with RV did not show an increase in either the expression of mucin genes or the number of goblet cells in the airway epithelium (data not shown).

**Figure 8:**
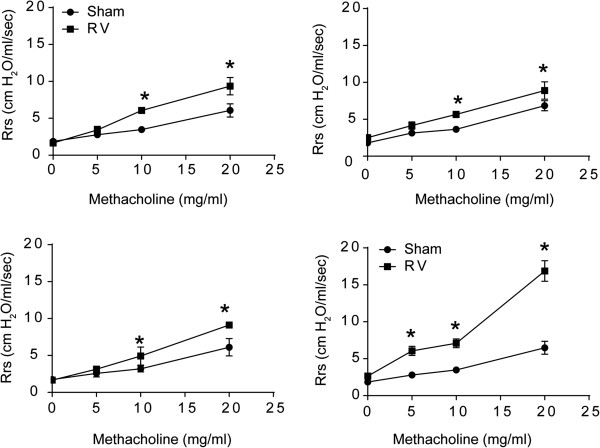
**Airways responsiveness to methacholine challenge following RV infection. RA, CS, HK-NTHi or CS/HK-NTHi-exposed mice were either infected with RV or equal volume of sham.** Four days later, total respiratory system resistance was measured by plethysmography. Data represent mean ± SEM from 3 mice per group (* different from respective sham-infected animals, p ≤ 0.05, two-way ANOVA).

RV-infected mice, irrespective of prior exposure to CS. NTHi, or combination of CS/NTHi, showed an increase in airways resistance in response to methacholine challenge over respective sham-infected mice (Figure 8A to 8D). However, CS/HK-NTHi-exposed mice infected with RV showed a much higher increase in airways resistance to methacholine challenge than similarly infected mice in other groups.

Taken together these results demonstrate that in addition to CS, bacterial products may also be required to induce COPD-like changes in both parenchyma and conducting airways of mice, and that CS/HK-NTHi-exposed mice are also more susceptible to subsequent RV infection.

## Discussion

Exacerbations in COPD patients are characterized by increased inflammation and increased lower respiratory tract symptoms requiring change in therapy such as treatment with antibiotics, steroids or antiviral drugs [33]. Exacerbations may be triggered primarily by viral or bacterial infections and are often associated with accelerated progression of lung disease. RV, which causes self-limiting infections in healthy individuals is responsible for the majority of virus-related exacerbations in patients with COPD [24,25], but the underlying mechanisms are not well understood. In this study, we developed a mouse model which displays phenotypic characteristics of COPD, including emphysema, mild but diffuse lung inflammation, and goblet cell metaplasia. We combined this model with our previously described mouse model of RV infection [22,34] and show that these mice show prolonged neutrophilic lung inflammation and airways obstruction, similar to that observed in mild COPD patients experimentally infected with RV [35], indicating the suitability of this model to elucidate mechanisms of COPD exacerbations.

The mouse model of COPD developed in this study supports our primary hypothesis that both CS and bacteria are required for development of COPD–like changes in mice and also render mice susceptible to viral infection. We demonstrate that although CS is the major risk factor in the development of COPD, mice exposed to CS alone for 8 weeks only develop mild lung inflammation and emphysema but not small airway disease which is consistent with previous observations [4,8-11]. In contrast, exposure of mice to a combination of CS and NTHi induces not only more pronounced emphysema and lung inflammation than mice exposed to CS alone, but also goblet cell metaplasia in the airways, which is one of the pathologic features of COPD. In addition, mice exposed to combination of CS/HK-NTHi also show heightened susceptibility to viral infection with further increases in lung inflammation and goblet cell metaplasia. Together, these findings suggest that this novel mouse model of COPD is not only useful in understanding the mechanisms of exacerbations, but also for delineating the mechanisms underlying progression of lung disease in COPD. However, it should be noted that continuous exposure to CS and/or intermittent infection with bacteria or virus may be required to maintain chronicity of the COPD-like changes in these mice.

NTHi is one of the more commonly isolated organisms from clinically stable COPD patients and also at exacerbations [23], and patients who are chronically colonized with NTHi show significantly more neutrophilic airway inflammation than those who are not colonized [15,36]. Further, repeated exposure to extracts of heat-killed NTHi causes airway inflammation in mice with cellular and cytokine profiles similar to COPD [18]. Finally, NTHi products increase mucin gene expression *in vivo* and *in vitro*[37]. These observations indicate that NTHi may contribute to the development of COPD particularly, small airway disease and lung inflammation. Based on these facts, we postulated that NTHi may synergize with CS to induce development of COPD-like features in mice which includes changes in both small airways and parenchyma. In the present study, we opted to use low-dose (5 × 10^6^ CFU) heat-killed NTHi to induce milder and sustained inflammation with minimal infiltration of neutrophils as opposed to acute neutrophil-dominated inflammation induced by live NTHi [27,28], to prevent extensive lung damage. The bacterial dose was chosen based on our preliminary experiments, in which mice were treated with heat-killed bacteria equivalent to 5 × 10^5^, 5 × 10^6^ or 5 × 10^7^ CFU once a week for 4 consecutive weeks and sacrificed 4 weeks after the last treatment. By morphology, mice treated with 5 × 10^5^ CFU did not show any detectable changes in the lungs and were very similar to untreated mice. In contrast, mice treated with 5 × 10^7^ CFU showed severe lung inflammation, consolidation of parenchyma and pronounced goblet cell metaplasia in both small and large airways. Mice treated with 5 × 10^6^ CFU showed mild to moderate lung inflammation with mild goblet cell metaplasia. In addition, we also found that two exposures to 5 × 10^6^ CFU instead of four exposures were sufficient to induce these changes in the lung. Combination of this HK-NTHi treatment with CS however, led to more pronounced lung inflammation and goblet cell metaplasia, increased mucin gene expression and also the development of emphysema. Interestingly these mice also showed thickening of airway epithelia, similar to the airway epithelial hyperplasia observed in COPD patients [38]. We also observed aggregates of inflammatory cells particularly in the peribronchiolar and perivascular areas resembling lymphoid aggregates [16], but the nature of these aggregates is yet to be determined. We speculate that these pronounced pathological changes in CS/HK-NTHi-exposed mice may be the result of exaggerated host innate immune responses to bacterial products, in the presence of CS.

Alveolar macrophages in the lungs play an important role in clearing bacteria and limit bacteria-induced inflammation. CS has been demonstrated to affect the function of alveolar macrophages by shifting their phenotype from M2 to M1 [39,40]. M1 macrophages respond to bacterial antigens such as LPS by producing relatively more inflammatory cytokines than M2 macrophages [41]. Although this is required for clearance of bacteria, sustained production of inflammatory cytokines may increase lung inflammation, emphysema and also induce goblet cell metaplasia [8,42]. Based on these observations, we speculate that CS-induced shift in macrophage phenotype may be one of the mechanisms by which CS synergizes with NTHi to increase expression of inflammatory cytokines and this in turn may lead to increased lung inflammation, emphysema and goblet cell metaplasia. Bacterial pathogens or their products other than NTHi may also induce COPD-like changes when combined with CS, but the magnitude of pathological changes may vary depending on the bacteria used. However, further studies are required to confirm this notion.

CS/HK-NTHi-exposed mice displaying the COPD phenotype were also found to be susceptible to subsequent infection with RV, a virus associated with a majority of viral-associated COPD exacerbations [35,43]. Four days after challenging with RV, CS/HK-NTHi-exposed mice despite showing minimally higher viral loads than similarly infected RA-, CS-, or NTHi-exposed mice, displayed sustained neutrophilia and lymphocyte infiltration, and increased expression of KC, MIP-2 and IP-10, which was not observed in mice from other groups. These changes were associated with progression in goblet cell metaplasia in small airways and increased expression of *Muc5AC* (mucin gene) and *Gob5*, a chloride channel that plays a role in mucin secretion [32]. These mice also showed sustained airways hyperresponsiveness to methacholine challenge. Previously, we have shown that airway epithelial cells isolated from COPD patients are more susceptible to RV infection despite expressing interferon and other antiviral genes [44]. This is probably due to increase in the number of goblet cells in COPD cell cultures, because previously goblet cells have been shown to be more permissive for RV infection [45]. Therefore it is plausible that CS/HK-NTHi mice which show increased numbers of goblet cells in their airways may be more susceptible to RV infection. In addition, exposure to CS may also support replication of virus and induce aberrant production of inflammatory cytokines. Consistent with this notion, acute exposure to CS was shown to increase production of pro-inflammatory cytokines and reduce expression of antiviral genes in response to RV infection in airway epithelial cells [46,47]. Such aberrant increases in inflammatory cytokines following RV infection may further enhance lung inflammation, goblet cell metaplasia and airways obstruction. However further studies are required to confirm this notion. These observations are consistent with human studies, in which mild COPD patients were shown to develop sustained lower respiratory tract symptoms and increased infiltration of neutrophil and lymphocyte in the lungs after experimental rhinovirus infection [35]. These changes correlated with decreases in lung function in these patients. Another noteworthy observation is that CS/HK-NTHi-exposed mice infected with RV also showed sustained increases in IP-10 levels, which has been proposed to be one of the biomarkers of COPD exacerbations [48,49]. These similarities in responses to RV infection between COPD patients and CS/HK-NTHi mice imply that this model may be useful in understanding the molecular mechanisms related to viral-associated COPD exacerbations and progression of lung disease.

## Conclusions

In summary, we show that CS synergizes with NTHi to cause a COPD-like phenotype in mice within a short period of time. As far as we know, this is the first mouse model of COPD to display innate immune responses to RV infection similar to that observed in COPD patients. Therefore we believe that this model could be used to elucidate the mechanisms of disease progression following acute exacerbations particularly that are associated with viral infections. This model may also be useful to model a chronic state of COPD, provided continuation of exposure to CS in addition to intermittent administration of bacterial or viral products.

## Abbreviations

COPD: Chronic obstructive pulmonary disease; RV: Rhinovirus; HK-NTHi: Heat killed nontypeable Haemophilus influenzae; CS: Cigarette smoke; RA: Room air; P-V: Pressure - volume.

## Competing interests

The authors declare that they have no competing interests.

## Authors’ contributions

SG, designed and conducted the experiments and analyzed the data; ATC and BK, provided technical support; PM and JMB, contributed intellectually; US, conceived and designed the study and wrote the manuscript. All authors read and approved the final manuscript.
